# ABD-Derived Protein Blockers of Human IL-17 Receptor A as Non-IgG Alternatives for Modulation of IL-17-Dependent Pro-Inflammatory Axis

**DOI:** 10.3390/ijms19103089

**Published:** 2018-10-09

**Authors:** Marie Hlavničková, Milan Kuchař, Radim Osička, Lucie Vaňková, Hana Petroková, Michal Malý, Jiří Černý, Petr Arenberger, Petr Malý

**Affiliations:** 1 Laboratory of Ligand Engineering, Institute of Biotechnology of the Czech Academy of Sciences, v. v. i., BIOCEV Research Center, Průmyslová 595, 252 50 Vestec, Czech Republic; marie.hlavnickova@ibt.cas.cz (M.H.); milan.kuchar@ibt.cas.cz (M.K.); lucie.vankova@ibt.cas.cz (L.V.); hana.petrokova@ibt.cas.cz (H.P.); michal.maly@ibt.cas.cz (M.M.); 2 Laboratory of Molecular Biology of the Bacterial Pathogens, Institute of Microbiology, Czech Academy of Sciences, v. v. i., Vídeňská 1083, 142 20 Prague, Czech Republic; osicka@biomed.cas.cz; 3 Laboratory of Structural Bioinformatics of Proteins, Institute of Biotechnology of the Czech Academy of Sciences, v. v. i., BIOCEV Research Center, Průmyslová 595, 252 50 Vestec, Czech Republic; jiri.cerny@ibt.cas.cz; 4 Department of Dermatology and Venereology, Faculty Hospital of Královské Vinohrady, Šrobárova 50, 100 34 Prague, Czech Republic; petr.arenberger@fnkv.cz

**Keywords:** binding protein, albumin-binding domain, cytokine, IL-17 receptor, combinatorial library

## Abstract

Interleukin 17 (IL-17) and its cognate receptor A (IL-17RA) play a crucial role in Th17 cells-mediated pro-inflammatory pathway and pathogenesis of several autoimmune disorders including psoriasis. IL-17 is mainly produced by activated Th-17 helper cells upon stimulation by IL-23 and, via binding to its receptors, mediates IL-17-driven cell signaling in keratinocytes. Hyper-proliferation of keratinocytes belongs to major clinical manifestations in psoriasis. To modulate IL-17-mediated inflammatory cascade, we generated a unique collection of IL-17RA-targeting protein binders that prevent from binding of human IL-17A cytokine to its cell-surface receptor. To this goal, we used a highly complex combinatorial library derived from scaffold of albumin-binding domain (ABD) of streptococcal protein G, and ribosome display selection, to yield a collection of ABD-derived high-affinity ligands of human IL-17RA, called ARS binders. From 67 analyzed ABD variants, 7 different sequence families were identified. Representatives of these groups competed with human IL-17A for binding to recombinant IL-17RA receptor as well as to IL-17RA-Immunoglobulin G chimera, as tested in enzyme-linked immunosorbent assay (ELISA). Five ARS variants bound to IL-17RA-expressing THP-1 cells and blocked binding of human IL-17 cytokine to the cell surface, as tested by flow cytometry. Three variants exhibited high-affinity binding with a nanomolar *K*_d_ value to human keratinocyte HaCaT cells, as measured using Ligand Tracer Green Line. Upon IL-17-stimulated activation, ARS variants inhibited secretion of Gro-α (CXCL1) by normal human skin fibroblasts in vitro. Thus, we identified a novel class of inhibitory ligands that might serve as immunosuppressive IL-17RA-targeted non-IgG protein antagonists.

## 1. Introduction

Interleukin 23/Interleukin 17 (IL-23/IL-17) pro-inflammatory axis plays a pivotal role in the pathogenesis of several chronic autoimmune diseases [1,2,3]. Heterodimer of a unique p19 subunit and a common p40 protein, both known as α- and β-subunits of IL-23 cytokine [4], is secreted by activated dendritic cells and macrophages, and stimulates the differentiation of naive Th cells into Th17 cell population. This action is mediated via interactions with its cognate receptor complex, formed by IL-23R and IL-12Rβ1 receptor units. Synergistic tethering of the IL-23 heterodimer to both receptor units leads to a receptor heterodimerization followed by a quaternary complex formation and triggering of Jak/Stat signaling cascade, involving Jak2, Tyk2, Stat1, Stat3, Stat 4, and Stat5 [5]. The activation pathway of Th17 cell stimulates transcription of several inflammatory genes, resulting in the secretion of inflammatory mediators including IL-17A/F cytokine [6,7].

IL-17 cytokine gene family consists of six members known as IL-17A, B, C, D, E, and F [8]. Among them, IL-17A and IL-17F have been described to closely associate with development of autoimmune diseases via interactions with a receptor complex formed by IL-17 receptor A and C [9]. After binding of IL-17A or IL-17F homodimers, or IL-17A/F heterodimer, to the IL-17 receptor complex composed of a heterodimer of IL-17RA and IL-17RC, Act1 associates with IL-17RA/RC through SEFIR domains and, via TRAF-6, promotes MAPK, NF-κB and C/EBP signaling [10]. IL-17A and F are mostly secreted by activated Th17 cells and γδT cells, but can be produced also by NKT cells or iLCs [11,12].

To target IL-23/Th17 pro-inflammatory cascade, several strategies have been developed. An attention has mostly been paid to blocking of an early step of the cascade by suppression of IL-23-mediated signaling in Th cells, thus suppressing the secretion of a cocktail of IL-17A, IL-22, and several chemokines by Th17 cells [13]. For psoriasis treatment, blocking of IL-17A cytokine or its receptor function on keratinocytes or skin fibroblasts is a valuable alternative. Originally, fully human monoclonal antibodies recognizing p40 subunit of the IL-23 cytokine were generated. While ustekinumab (Stelara) [14,15], a product of Janssen Biotech Inc., has been proved to be safe and already reached the market, another one, Briakinumab, was withdrawn due to an increased risk of cardiovascular side effects [16,17]. The strategy for modulation of IL-23/Th17 axis has been changed by focusing on p19 protein with aim to increase the specificity and safety of the drugs. Currently, there are several fully human or humanized monoclonal antibodies targeting the p19 in the development. Those include Guselkumab [18,19], Tildrakizumab (MK-3222) [20,21], and Risankizumab (B1655066) [22]. 

Alternatively, several neutralizers of downstream pathways of the IL-23/Th17 inflammatory cascade have been developed. Among them, fully human monoclonal antibody secukinumab (Cosentyx), a product of Novartis International AG, targeting human IL-17A cytokine, is available for treatment of psoriasis, ankylosing spondylitis, and psoriatic arthritis [23,24,25]. Fully human monoclonal antibody ixekizumab (Taltz) by Eli Lilly and Co., blocking IL-17A-mediated signaling, is approved for treatment of moderate-to-severe forms of plaque psoriasis and psoriatic arthritis [26]. Brodalumab (Siliq, Kyntheum), a human monoclonal antibody-based antagonist of IL-17 receptor A developed by Astra Zeneca, demonstrated an excellent efficacy in treatment of moderate-to-severe forms of plaque psoriasis [27,28]. In addition to that, soluble mutants of human IL-17A receptor were also developed and they exhibited a promising therapeutic effect in mouse model of psoriasis [29].

While monoclonal antibody-based biologics still represent a major line in drug development, non-immunoglobulin binding proteins derived from small protein domain scaffolds, generated by a directed evolution approach, attract attention as next generation therapeutics [30,31]. Small binding proteins selected from highly complex combinatorial libraries to any chosen target can be further engineered for desired specificity and binding affinity by in silico approaches. In contrast to antibodies, they lack disulfide bridges, can be produced in vitro and easily modified by molecular and gene-fusion approaches. Due to a small size, they exhibit an excellent tissue penetration and, in combination with transdermal delivery systems, they can be useful for development of topically-administrated drugs. Albumin-binding domain (ABD) of streptococcal protein G belongs to the smallest protein domain scaffolds that have been successfully verified for the generation of highly complex combinatorial libraries. This scaffold of 46 amino acids is formed by a three-helix bundle (pdb id 1GJT, residues 20–65) which provides three surfaces amenable to randomization [32,33,34]. As the parental non-mutated wild-type ABD scaffold does not contain cysteine residues, it can be easily refolded into a functional state, supporting its binding specificity. As the critical residues for a high-affinity binding to human serum albumin (HSA) are located on the second and third helices, we selected this surface for the randomization of 11 residues, thus disrupting the original HSA binding sites and providing a library of a theoretical complexity 2 × 10^14^ protein variants. The validity of the library concept has already been demonstrated by the generation of ABD-derived binders targeting human interferon-γ with a sub-nanomolar affinity [32], by immunosuppressive protein blockers of human IL-23 receptor [35], by development of ABD-derived binders of human prostate specific protein 94 (PSP94, MSMB) [36] and by Shiga toxin 1B subunit-specific binders [37], and recently by immunomodulatory binders of human IL-23 cytokine [38]. 

In this work we used the ABD-derived highly complex combinatorial library to generate a unique collection of high-affinity binding proteins targeting the human IL-17 receptor A. We selected 7 representatives of the found sequence families and demonstrated that 5 of them interact with a soluble IL-17RA-IgG chimera and bind to IL-17RA-expressing THP-1 cells. In correlation to a predicted blocking function by in-silico docking, these variants inhibited binding of IL-17A cytokine to the recombinant IL-17RA and exhibited an immunosuppressive potential demonstrated on normal human skin fibroblast in vitro. We, therefore, contribute to the development of novel non-IgG immunomodulatory agents that can be useful in targeting of IL-23/Th-17 pro-inflammatory axis as unique IL-17RA antagonists.

## 2. Results

### 2.1. Molecular Assembly and Production of Recombinant IL-17RA

In our previous work [35] we demonstrated that the extracellular moiety of the human IL-23 receptor gene can be successfully produced as a refolded bacterial protein, which can serve as a target for the selection of protein binders from a highly complex combinatorial library. Therefore, we used a similar approach here and cloned the cDNA sequence coding for an extracellular part of the human IL-17RA (IL-17RAex), consisting of two fibronectin-type III domains (amino acid residues 33–320), into the bacterial expression vector pET-28b. This recombinant plasmid was used for IL-17RAex production in the *E. coli* SHuffle strain upon the optimization of the induction at 18 °C. Under these conditions, the major amount of the IL-17RAex protein was produced in an insoluble fraction from which the protein was purified by affinity chromatography on a Ni-NTA-agarose column under denaturing conditions. This urea-containing solution was diluted in a coating buffer, followed by refolding on Polysorp plate using several washing steps with PBS buffer. The significant amount of IL-17RAex was also present in a soluble fraction from which the protein was purified by Ni-NTA chromatography under native conditions. The identity of the purified IL-17RAex variants was verified on SDS-PAGE, as seen in Figure S1A, and by Western blot using anti-IL-17RA polyclonal antibody. Refolded IL-17RAex product, similarly to the soluble product, bound human IL-17A cytokine in enzyme-linked immunosorbent assay (ELISA) as seen in Figure S1B. We, therefore, used the soluble IL-17RAex as well as the refolded product as target proteins for the selection of IL-17RA-targeted protein binders. 

### 2.2. Selection of ABD-Derived Variants Producing IL-17RA-Targeted Proteins

**Figure 1:**
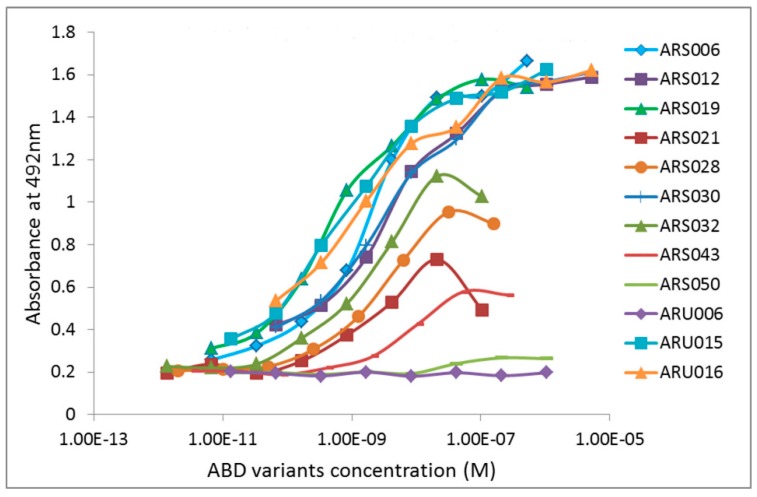
Screening of the interleukin 17 cognate receptor A (IL-17RA)-binding variants in enzyme-linked immunosorbent assay (ELISA). Bacterial cell lysates of individual ARS and ARU clones were screened for binding to the immobilized recombinant IL-17RA receptor. The binding proteins were produced in the form of in vivo biotinylated ARS/ARU-TolA-AVI fusion proteins and their binding to IL-17RA was visualized by streptavidin-HRP conjugate.

In our previous studies [32,35,36], we used only one recombinant product as a target for the selection of high-affinity binders. In this study, we decided to independently select ligands against the IL-17RAex protein purified either from the soluble fraction or from the insoluble fraction, and to investigate whether ribosome display will generate collections of IL-17RA binding proteins of the same sequence identity. After 5-round ribosome display with the protein purified from the soluble fraction, we generated a plasmid library carrying ABD-derived sequences encoding IL-17RAex-targeted protein variants that were called ARS ligands. Similarly, using the IL-17RAex protein purified from the insoluble fraction, we generated a plasmid library encoding a collection of protein binders, called ARU binders. Both libraries were screened for the identification of the best binding candidates using ELISA. For this screening, ARS and ARU variants were produced in the form of in vivo biotinylated His_6_-ARS/ARU-TolA-AVI fusion proteins, biotinylated through the C-terminal AviTag sequence (GLNDIFEAQKIEWHE) in BirA-positive *E. coli* host cells. An example of the screening of the ARS and ARU variants is presented in Figure 1. Production of 40 kDa ARS/ARU proteins of the selected candidates was verified by Western blot using an anti-His-tag antibody.

**Figure 2:**
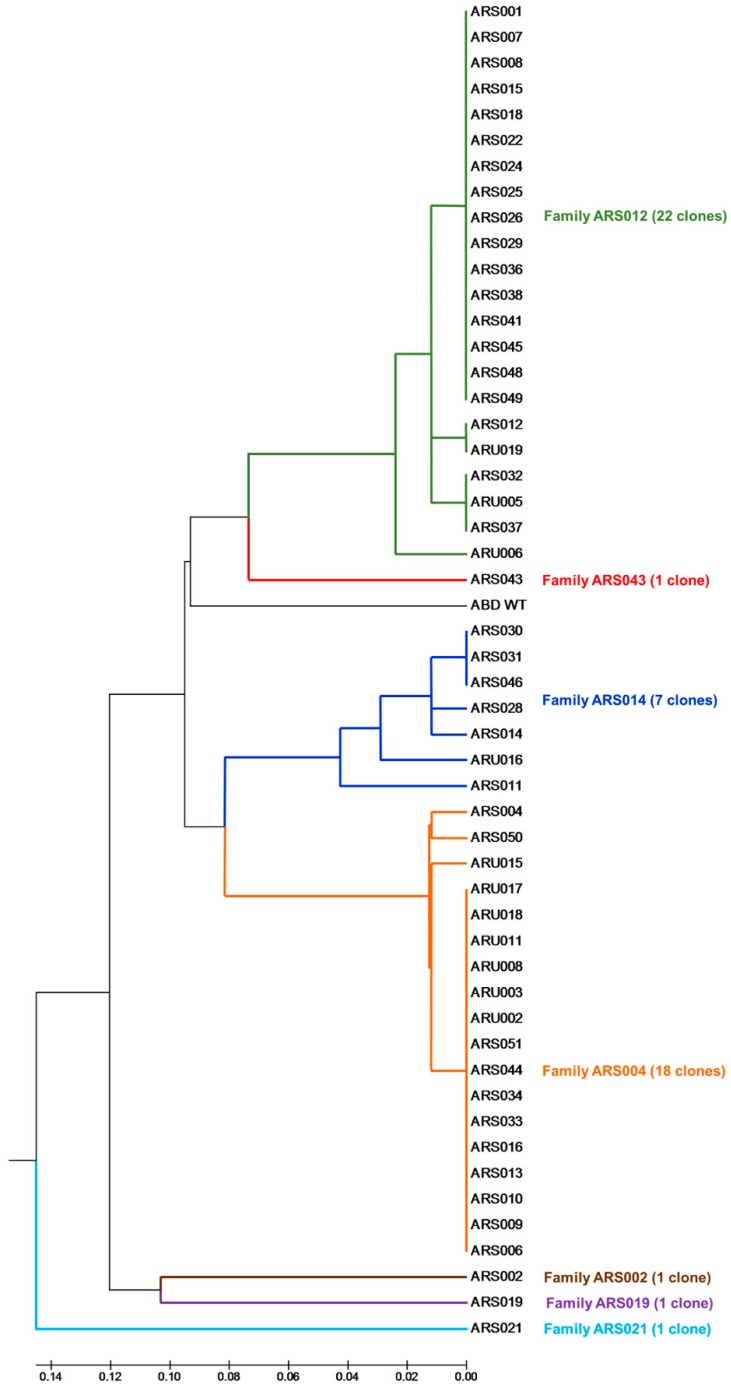
Comparison of a sequence similarity of the selected ARS and ARU clones. Similarity tree of polypeptide sequences of 51 analyzed ARS and ARU variants that were targeted to the recombinant extracellular IL-17RA receptor and selected by ribosome display. Sequence analysis of the ARS/ARU binders revealed 17 unique sequence variants that were clustered into 7 sequence families. For a similarity analysis, only the sequences between residues 20 and 46 were compared, as the N-terminal amino acid positions 1–19 were non-randomized.

Following the experimental approach used previously for IL-23 receptor-targeted REX and IL-23 cytokine-targeted ILP binder selections [35,38], we identified 67 clones of ARS/ARU-producing variants that resulted from screening of both types of plasmid libraries. Plasmid DNA coding for all these clones was analyzed by sequencing, providing a final collection of all DNA sequences. Comparison of sequence similarity among all 67 tested clones and the parental non-randomized ABD was performed and this result is shown as a phylogenetic tree of 51 clones in Figure 2 (16 missing members are only sequentially redundant clones of ARS001). We found that representatives of ARU variants matched well to variants of the ARS collection, represented by the sequence identity or a high level of similarity. Contrary that, some of the ARS variants were unique (ARS002, ARS019, ARS021) or did not overlap with ARU sequences at a high similarity level (ARS043). As the ARS library represents a more complex collection, we selected ARS variants as representatives for 7 identified sequence families as seen in Figure 2. Those are ARS004 and ARS012 with many redundant clones, ARS014 with a limited sequence variability, and ARS002, ARS019, ARS021, and ARS043, each identified by one unique representative. These 7 ARS variants were selected as major candidates for further analysis.

### 2.3. Characterization of Binding Properties of the Selected ARS Variants 

**Figure 3:**
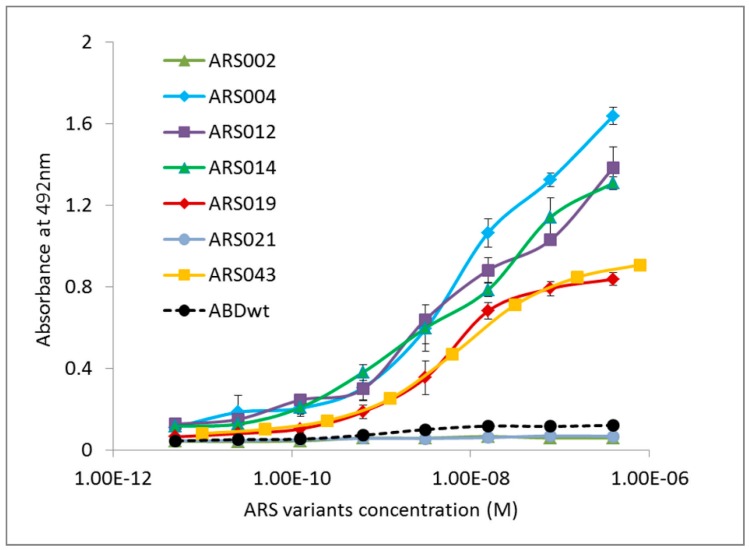
Binding of the selected representatives of ARS sequence families to the immobilized human IL-17RA-IgG chimera in ELISA. Purified binding proteins were produced in the form of in vivo biotinylated His_6_-ARS-TolA-AVI fusion proteins. Binding to IL-17RA-IgG was visualized by streptavidin-HRP conjugate. Each point represents the mean value ± standard deviation (SD).

Representatives of the ARS sequencing families were produced in the form of fusion proteins containing a polyhistidinyl tag at the N-terminus and a 305 residues long helical TolA spacer protein and an Avitag consensus sequence as a target for in vivo biotinylation at the C-terminus (His_6_-ARS-TolA-AVI). The fusion proteins were purified using affinity chromatography on a Ni-NTA agarose column and tested for binding to the IL-17 receptor. As all identified ARS and ARU clones were originally selected via targeting to the recombinant IL-17RAex protein expressed in *E. coli*, we investigated whether the selected ARS binding proteins can recognize a soluble glycosylated product of the human IL-17RA produced in the form of IL-17RA-IgG chimera. As shown in Figure 3, the ARS004, ARS012, ARS014, ARS019 and ARS043 variants in the form of His_6_-ARS-TolA-AVI fusion proteins bound to the IL-17RA-IgG chimera, while no binding was observed with the ARS002 and ARS021 variants, similarly as the parental non-randomized ABD wild-type protein. These binding variants were, therefore, identified as major candidates for further studies.

**Figure 4:**
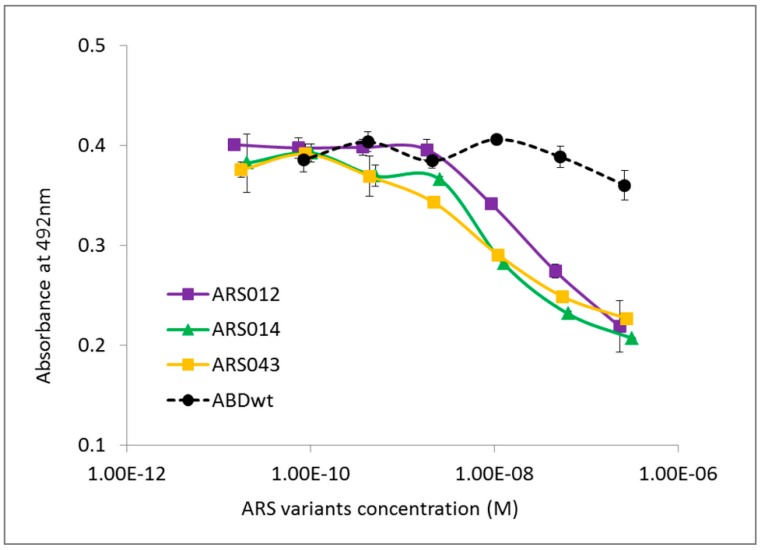
ARS ligands compete with IL-17A cytokine for binding to the human IL-17RA-IgG receptor chimera. The IL-17RA-IgG chimera was immobilized on an ELISA plate and serially diluted inhibitory His_6_-ARS-TolA-AVI ligands were used to compete for binding with 10 nM IL-17A. Bound IL-17A was detected with anti-IL-17A polyclonal antibody in combination with secondary anti-IgG-HRP conjugate. His_6_-ABDwt-TolA-AVI served as a negative control. Error bars represent standard deviations (SDs).

To verify this fact, we performed the same competition ELISA experiment using the soluble IL-17RA-IgG chimera as a target. We tested all the selected ARS variants that were identified as binders of the IL-17RA-IgG chimera as seen in Figure 3. As presented in Figure 4 and Figure S2B, ARS004, ARS012, ARS014, ARS019, and ARS043 suppressed binding of IL-17A to the chimera, suggesting a blocking potential of these variants.

Protein sequences of the five ARS protein variants that bound to the soluble IL-17 receptor chimera and exhibited a blocking potential are presented in Table 1. 

In our previous studies we demonstrated that the 305 residues long helical TolA protein served as a solubility- and stability-supporting moiety of the 40 kDa fusion protein, which did not interfere with the binding specificity of the tested ligands [32,36,38]. To demonstrate this fact in this study, we constructed short forms of selected ARS variants lacking the TolA protein sequence. These short variants of 8.3 kDa were produced as soluble His_6_-ARS-AviTag in vivo biotinylated proteins, as seen in Figure S3A, and their binding to IL-17RA was confirmed by ELISA, shown in Figure S3B. We further used these short ARS protein forms to verify an inhibitory function of the long TolA-containing ARS forms as it has been demonstrated in Figure S2 and Figure 4. Therefore, we performed a competition ELISA experiment in which the purified short ARS variants competed with IL-17A cytokine for binding to IL-17RA. As presented in Figure S4, the short ARS variants, presented as examples of ARS012s, ARS014s and ARS043s, inhibited binding of human IL-17A, thus supporting previous conclusions done using the long His_6_-ARS-TolA-AVI variants. In contrast to this, a short ARS002s protein did not exhibit an inhibitory potential shown in Figure S2 for the long His_6_-ARS-TolA-AVI fusion protein.

### 2.4. Binding of the ARS Variants to Human Cells

**Figure 5:**
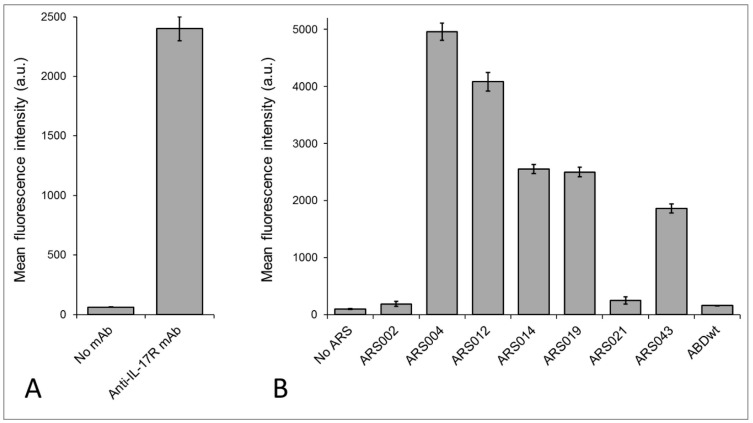
Binding of the ARS variants to human THP-1 cells. (**A**) The expression of IL-17RA on the surface of THP-1 cells was confirmed by anti-IL-17RA antibody. (**B**) For binding assay, 2.5 × 10^5^ THP-1 cells were incubated with in vivo biotinylated His_6_-ARS-TolA-AVI proteins or His_6_-ABDwt-TolA-AVI negative control (10 µg/mL) for 30 min at 4 °C. The cell-bound proteins were stained with streptavidin-PE for 30 min at 4 °C and analyzed by flow cytometry. Each bar represents the mean value ± SD of three independent experiments.

Human uninduced THP-1 myelomonocytic cells are known to express the IL-17 receptor [39]. This was confirmed by flow cytometry upon staining of THP-1 cells with anti-human IL-17RA antibody as seen in Figure 5A. Thus, this cell line was used to determine, which of the seven selected representatives of the ARS sequence families bind to the IL-17RA. As shown in Figure 5B, the variants ARS002 and ARS021 exhibited only a negligible binding to THP-1 cells, which is in a good agreement with the ELISA results, where these two proteins did not significantly bind the coated IL-17RA-IgG chimera, shown in Figure 3. In contrast to that, all ARS variants found to be binders of the IL-17RA-IgG chimera, as seen in Figure 3, substantially bound to IL-17RA-expressing THP-1 cells as shown in Figure 5B.

The ARS variants confirmed to bind to THP-1 cells were used for a cell-surface competition experiment in which the ARS ligands competed with the human IL-17A cytokine for binding to IL-17RA-expressing cells. As shown in Figure S5, all five ARS variants substantially reduced binding of IL-17A to the surface of THP-1 cells.

### 2.5. Determination of Binding Kinetics and Affinities of the ARS Variants Using Human Cells

**Figure 6:**
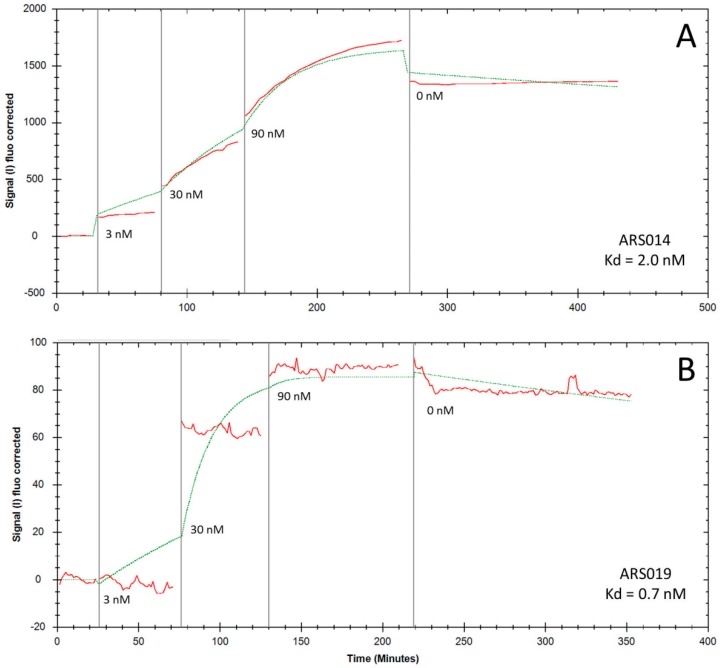
Binding of ARS014 (panel (**A**)) and ARS019 (panel (**B**)) to HaCaT cells tested by LigandTracer Green Line system. For binding assay, 10^6^ cells were plated overnight on Petri dish and the next day, in vivo biotinylated His_6_-ARS-TolA-AVI proteins were added into medium and incubated gradually at three different concentrations. Cell-bound proteins were stained with streptavidin-APC conjugate and the measured binding curve (red line) was analyzed using the TraceDrawer software (fitted curve, green line). Analysis of the binding affinities and rate-off kinetics indicated K_d_ values for ARS014 and ARS019 to be 2 nM and 0.7 nM, respectively.

To investigate binding parameters of the five ARS variants that bound to human THP-1 cells, as seen in Figure 5, we used adherent IL-17RA-expressing human keratinocyte HaCaT cells [40]. Determination of binding affinities and rate-off kinetics in a real-time fluorescent mode was done using Ligand Tracer Green Line system upon detection of the bound ARS proteins to the cell surface by secondary Streptavidin-APC conjugate. To get binding curves, we used three concentrations of each ARS protein (3 nM, 30 nM, and 90 nM) and rate-off kinetics were evaluated after a saturation by measuring in medium lacking the ARS binders. As shown in Figure 6, a very slow release of the ARS014 and ARS019 variants from the cell surface was observed. This indicates high-affinity binding and corresponds to values calculated by Trace Drawer software as *K*_d_ = 2 nM for ARS014 and 0.7 nM for ARS019, respectively. Using this approach, we also estimated *K*_d_ value for ARS004 (1.2 nM), ARS012 (35 nM) and ARS043 (32 nM), respectively.

### 2.6. Thermal Stability of the ARS Binding Proteins

**Figure 7:**
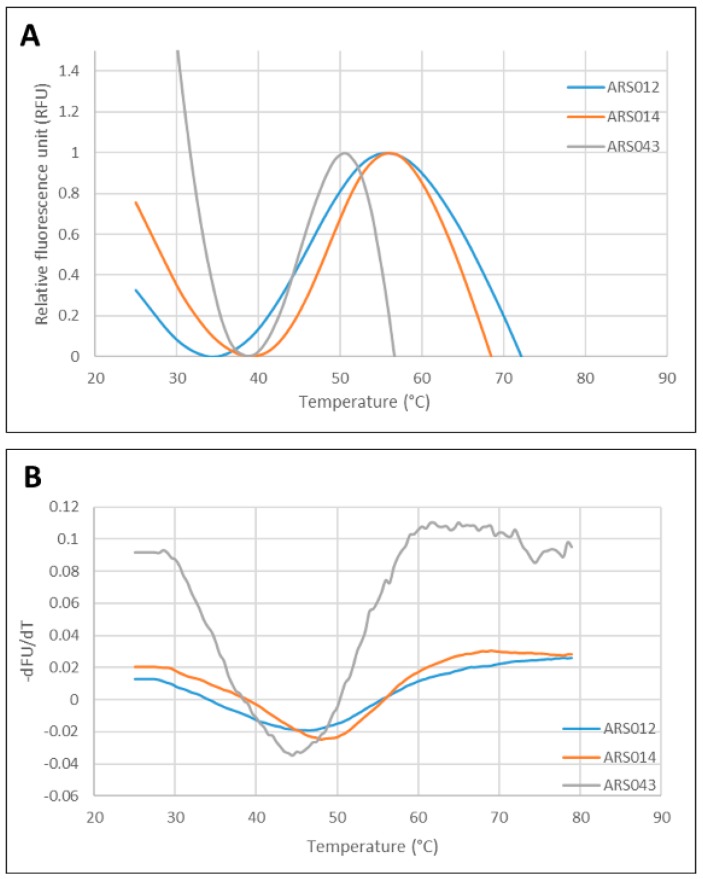
Analysis of thermal stability of the selected ARS binders. (**A**) Normalized thermal melting fluorescence curves of the His_6_-ARS-TolA-AVI binders. (**B**) First derivative of fluorescence versus temperature of curves shown in the panel **A**. The melting point is given as the lowest point of the curve. All measurements were done in duplicate and averaged. Identified temperature melting (*T*m) points are as follows: ARS012 *T*m 45.5 °C; ARS014 *T*m 48 °C; ARS043 *T*m 44.5 °C.

For the analysis of thermal stability of the most promising ARS variants, we used the fluorescently-based thermal-shift assay using His_6_-ARS-TolA-AVI fusion proteins. Melting temperatures (*T*m) found for the ARS012, ARS014 and ARS043 variants are presented in the Figure 7. Thermal stability of the ARS019 and ARS 004 binding proteins was not determined, as the used experimental approach failed for these particular protein variants. In our previous studies [32,35,38] we measured the thermal stability of the parental ABD-wild type protein using the same approach and provided the *T*m value 58 °C. Thus, our results suggest that the randomization of mutable residues of the ABD domain significantly affected the basic stability of the scaffold structure in the case of ARS012, ARS014, and ARS043.

### 2.7. Modeling of ARS-IL17RA Interactions

**Figure 8:**
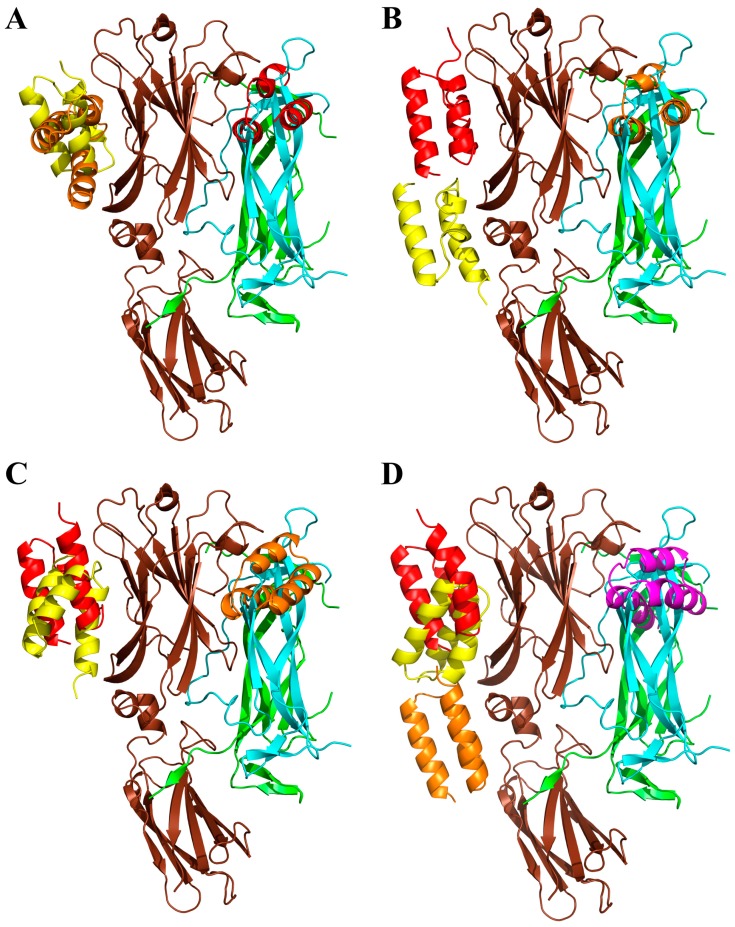
Modeling of ARS/IL-17RA interactions by docking. Summary of the first three poses of the ARS004 (**A**), ARS012 (**B**), ARS019 (**C**), and four poses of the ARS043 (**D**) binding to the complex of IL-17RA (brown) and dimer of human IL-17A (green/cyan), in decreasing predicted order of probability demonstrated in red (the most probable), orange, yellow and magenta, respectively.

To gain a structural insight of the ARS-IL-17RA interactions, we performed modeling of the IL-17RA receptor in a complex with particular ARS variants. Therefore, we prepared homology models of the ARS binding proteins based on a three-helix bundle structure of the ABD. Then, we identified probable binding modes of the ARS binders found on the IL-17RA receptor by docking. For the ARS004, ARS012, ARS019, and ARS043 variants, binding modes are shown in Figure 8. In the case of the ARS004 variant, the most probable binding mode predicted by docking was located in the distal IL-17RA receptor domain, which is crucial for recognition by IL-17A cytokine, as shown by the crystal structure of the IL-17A/IL-17RA complex. This is well documented in Figure 8A where ARS004 binding protein clearly overlaps with IL-17A cytokine domains. For the variants ARS012 and ARS019, the same binding area on the IL-17RA domain as that found for ARS004 was predicted as the second most probable binding mode, as seen in Figure 8B,C. The same binding area on the receptor was identified by docking of the ARS043 binder as the 4th most probable binding mode, as shown in Figure 8D. These results correspond to data of competition ELISA tests where inhibitory effects of these ARS variants were demonstrated as seen in Figure 4, Figures S2 and S4.

**Figure 9:**
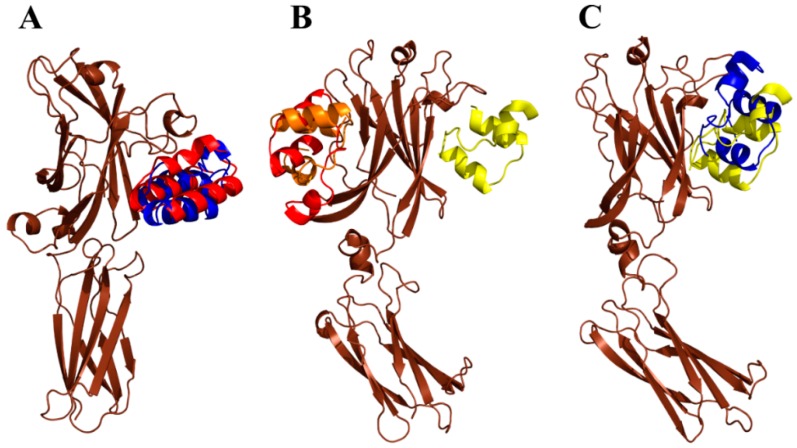
Structural effect of amino acid alterations in the ABD scaffold. (**A**) The most probable pose of the ARS004/IL-17RA interaction obtained by flexible side chain docking (red, see also Figure 7A) compared to the structure relaxed by 100 ns molecular dynamics simulation (**blue**). (**B**) Modeling of the ARS014/IL-17RA interaction by docking. The initial geometry of ARS014 was obtained from a 1 µs molecular dynamics simulation. Summary of the first three poses of ARS014 binding to the IL-17RA (**brown**) is shown in decreasing predicted order of probability demonstrated in red (the most probable), orange, and yellow, respectively. (**C**) The third most probable pose (**yellow**) of the ARS014/IL-17RA interaction compared to the structure relaxed by 100 ns molecular dynamics simulation (**blue**).

Interestingly, docking of the ARS014 variant did not predict any binding into the identified common binding area of the receptor among 10 the most probable binding modes. However, this finding does not correspond to a demonstrated inhibitory effect of this ARS variant found in the competition ELISA tests, as seen in Figure 4, Figures S2 and S4. The ARS014 variant differs from other analyzed ARS proteins by a double deletion in positions 35–36 of the ABD sequence as shown in Table 1. This could lead to an increased probability that the used homology model does not reflect the equilibrium structure of the binding protein. Therefore, we performed further modeling using molecular dynamics simulations looking for structural consequences of the specific combinations of amino acid alterations. Molecular dynamics simulation of the ARS004 indicates that the structure of this variant remains similar to that of an initial homology model keeping the three-helix bundle conformation of the parental ABD template. This structure we used for molecular docking to the IL-17RA. This analysis predicted the same binding mode as found for the initial unrelaxed homology model. To validate the docking prediction, we studied a possible rearrangement of the ARS004/IL-17RA complex by means of molecular dynamics. Our results supported stability of the binding mode, which was predicted by docking as seen in Figure 9A. Following this approach, we performed the molecular dynamics of the ARS014 binding protein and identified a significant structural change of the C-terminal helix resulting in a perpendicular orientation of the helix in contrast to the initial parallel orientation found in other analyzed ARS variants. This structure was used for docking to the IL-17RA. This modeling approach led to the identification of the third most probable binding mode occupying the common binding area of the IL-17RA, as seen in Figure 9B. Molecular dynamics simulation of the ARS014/IL-17RA complex revealed that both interacting interfaces undergo a mutual structural rearrangement, shown in Figure 9C, leading to a larger interacting area resulting in an increased binding affinity (*K*_d_ 2 nM) as measured experimentally and presented in Figure 6A.

### 2.8. Immunomodulatory Potential of the ARS Binding Proteins

**Figure 10:**
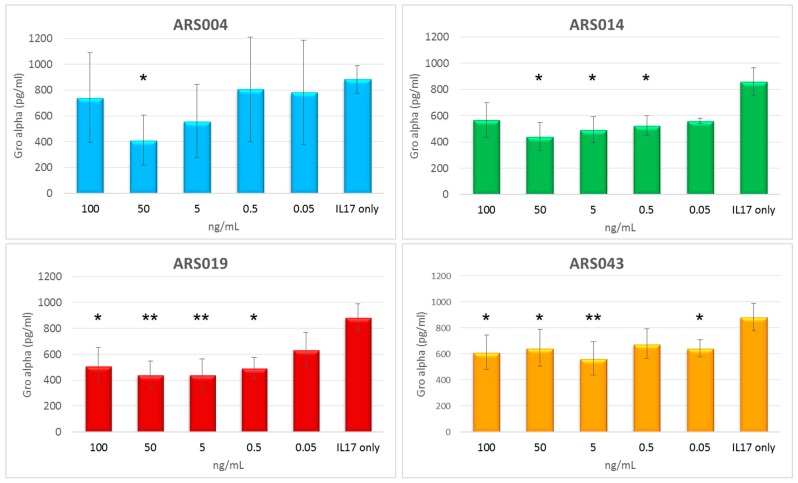
Test of immunomodulatory effect of the ARS ligands. The His_6_-ARS-TolA-AVI fusion proteins were added in different concentrations to medium containing IL-17A cytokine. As a control, medium with cytokine but without the ARS proteins was used (IL17 only). After 24 h, levels of Gro-α (CXCL1) secreted by CCD-1070Sk cells were measured by ELISA. Each bar represents the mean value ± SD of four (ARS019 and ARS043) or two (ARS004 and ARS014) independent experiments. Significant differences between mean values of the control (IL17 only) and the samples are shown (*, *p* < 0.05; **, *p* < 0.01; analysis of variance (ANOVA)).

To study an immunomodulatory potential of the particular ARS protein variants, we used normal human skin fibroblast cell line CCD-1070Sk, which has been previously described to be suitable for testing of the immunosuppressive potential of soluble IL-17 receptor mutants [29]. As a first step, we measured secretion of Gro-α (CXCL-1) released by CCD-1070Sk cells upon the activation by IL-17 cytokine using ELISA. This chemokine was significantly present in the cell medium supernatants after 6 h of activation by IL-17A, but the most suitable time period was found to be between 15 and 30 h after the activation, where levels of the secreted protein reached maximum values as seen in Figure S6. Therefore, we activated CCD-1070Sk cells by IL-17A for 24 h in the presence or absence of the five selected ARS protein variants and measured levels of secretion of the Gro-α in cell supernatants. Results of these experiments summarized in Figure 10 suggested the immunosuppressive potential for ARS004, ARS014, ARS019, and ARS043, where the inhibition of secretion of the chemokine was demonstrated. Contrary that, ARS012 protein binder did not exhibit any inhibitory function.

## 3. Discussion

To interfere with IL-17-driven immunomodulation via IL-17RA-mediated cell signaling, we used a non-immunoglobulin approach. Recently we demonstrated that albumin-binding domain-derived library was successfully used for the generation of immunosuppressive binding proteins targeted to IL-23 cytokine [38] and for the development of IL-23R-targeted antagonists [35]. In these studies, we used our own concept of the highly complex ABD-derived library in which randomization of 11 residues of helix 2 and helix 3 of the ABD scaffold disrupts a parental high-affinity binding to human serum albumin [32] but also eliminates a set of crucial residues of the known human T- and B-cell epitopes carried by the parental non-mutated ABD wild-type protein [38,41]. 

To target the IL-17RA, soluble as well as refolded bacterial products of the extracellular receptor domains were used as capture proteins for ribosome display selection. We demonstrate that both protein variants can be used for the selection of high-affinity binders. Interestingly, the ARU collection of binders of the refolded IL-17 receptor provided variants identical, or of a high sequence similarity (1–2 amino acid alteration), to those found in the ARS protein library generated with the soluble IL-17RA. Yet the ARS library of selected variants seems to be more complex by the presence of several unique clones absent in the ARU collection. A high correlation between both libraries is rather surprising and underlines the high efficiency of ribosome display selection. Such comparison of independent selections of non-IgG binding proteins targeted to different forms of the same protein target has not been described for other known protein scaffold libraries. 

Among 7 sequentially unrelated families identified by alignment and clustering, five of them are of a high interest as those represent blockers of cell-surface IL-17RA. Among them, ARS014 group is important as this family of 7 variants is formed by 5 different protein forms. As ARS014 representative belongs to the best blocking candidates, each member of this group should be analyzed for its immunosuppressive potential. Contrary that, family of ARS004 of 18 representatives contains only 4 sequence variants, as the majority of clones is redundant. Even higher redundancy was found in the ARS012 sequence group, while families of ARS019 and ARS043 are formed by a single variant. Interestingly, only these two family representatives lack deletions in the ABD sequence, as the ARS004 and ARS012 miss the residue 23N and ARS014 is characterized by a double 35/36 deletion gap followed by the residue 37P. This unique combination alters the parental ABD three-helix bundle scaffold to a structurally more variable moiety that complicates modeling by docking. Paradoxically, this relaxed conformation may well accommodate to its receptor counterpart, providing an increased ligand stability, supported also by the highest thermal stability among the five ARS blocking candidates, and by a high binding affinity. However, ARS ligands stability is, in a broader context, significantly lower in comparison to ILP or REX immunosuppressive binders [35,38]. This might be due to the presence of proline residues found in the randomized positions of all five blocking ARS variants, thus changing the local conformation of the protein backbone or breaking the optimal three-helical structure.

Prediction of binding areas on the IL-17RA surface by docking ascribed a probable blocking potential to all five ARS ligands, yet for the ARS014 variant only upon the relaxation of its structure. The ARS004 protein was predicted as the most promising inhibitor of the cytokine IL-17A binding to its cognate receptor. This correlates with competition ELISA data but it is also strongly supported by cell-surface competition binding test on THP-1 cells and by the strong binding affinity estimated by LigandTracer on HaCaT keratinocytes. Slow dissociation of the ARS004 from the cell-surface receptor significantly contributes to the observed immunosuppressive effect on normal human IL-17A-activated skin fibroblasts. These cells were very efficiently suppressed in secretion of Gro-α by the presence of ARS014 and ARS019 variants in cell medium. This fact correlates very well with a slow dissociation of both proteins blockers from the HaCaT cell surface. In addition, an inhibitory function for both these variants was predicted by docking as highly probable, i.e., as the second (ARS019) and third (ARS014) most probable binding areas overlapping with the region known to be recognized by IL-17A [42]. Interestingly, ARS012 competed with IL-17A in vitro as shown by ELISA, as seen in Figure 4, and THP-1 cell-surface competition binding assay, shown in Figure S5, while its immunosuppressive potential has not been demonstrated in normal human skin fibroblasts. As ARS012 inhibitory potential on activated CCD-1071Sk cells is not conclusive and ARS043 exerts only a mild suppressing potential, variants ARS004, ARS014 and ARS019 are the best candidates for further development of IL-17R antagonists. However, their anti-inflammatory potential needs to be yet verified in vivo.

Although the IL-23/Th17 pro-inflammatory axis is closely linked to development of psoriasis, psoriatic arthritis and rheumatoid arthritis, a substantial role for IL-17A/F cytokine and IL-17RA in intestinal inflammation such as inflammatory bowel disease and Crohn´s disease has been documented [43,44]. Recently, we described the generation of unique strains of *Lactococcus lactis* that can produce and secrete ABD-derived ILP proteins, blocking the function of human IL-23 cytokine [45]. The ILP-producing *L. lactis* cells can be used as in vivo tolerable probiotic bioreactors for the production and delivery of anti-inflammatory agents during the intestinal inflammation. In this context, a novel class of anti-IL-17RA-targeted ABD-derived blockers can be used for the development of ARS-producing *L. lactis* strains useful for in vivo suppression of experimentally induced colitis. 

Anti-IL-17A inhibitors play a substantial role in development of anti-cancer drugs for colorectal cancer. Elevated levels of IL-23, IL-17, and IL-6 in early stages of colorectal cancer have been associated with adverse prognosis and more aggressive disease [46]. IL-17A is also associated with development of skin cancer via STAT3-mediating signaling in tumor and stromal cells, thus stimulating the penetration of myeloid cells into tumor environment [47,48]. Generation of non-IgG protein blockers of IL-17A-mediated signaling is, therefore, a valuable alternative in anti-cancer drug development.

## 4. Materials and Methods 

### 4.1. Antibodies, Recombinant Proteins and Detection Agents

Anti-human IL-17R rabbit polyclonal antibody (H-168 clone) was purchased from Santa Cruz Biotechnology Inc., Dallas, TX, USA. Goat anti-rabbit IgG-HRP conjugate was obtained from Abcam plc., Cambridge, UK. The monoclonal anti-poly-histidine-HRP antibody produced in mouse was obtained from Sigma-Aldrich, St. Louis, MO, USA. Rabbit polyclonal to human IL17A was obtained from Abcam plc., Cambridge, UK. Cy5-conjugated goat anti-rabbit IgG F(ab´)_2_ fragment was obtained from Jackson ImmunoResearch Laboratories, West Grove, PA, USA.

Recombinant human IL-17 RA/IL-17 R Fc Chimera was obtained from R&D Systems, Minneapolis, MN, USA. Human IL-17A was purchased from Cell Signaling Technology, Danvers, MA, USA. Streptavidin-phycoerythrin was purchased from eBioscience, San Diego, CA, USA. Streptavidin-HRP conjugate was obtained from Thermo Scientific, Rockford, IL, USA. 

### 4.2. Cell Lines and Growth Conditions

Cell lines used in the study were human acute monocytic leukemia cell line THP-1 (ATCC^®^ TIB-202), normal human skin fibroblast CCD-1070Sk (ATCC^®^ CRL-2091, ATCC, Manassas, VA, USA.), and a spontaneously transformed aneuploid immortal keratinocyte cell line from adult human skin HaCaT (330493, CLS Cell Lines Service GmbH, Eppelheim, Germany). All cell lines were grown in DMEM medium (Sigma-Aldrich, St. Louis, MO, USA) supplemented with 10% fetal calf serum (FCS) (GIBCO, Grand Island, NY, USA) and antibiotic antimycotic solution (ATB) (Sigma-Aldrich, St. Louis, MO, USA).

### 4.3. Molecular Assembly and Production of Recombinant IL-17RA

For IL-17A receptor (accession #: BC011624) cloning, plasmid DNA (Source BioScience, 4053746) was used. DNA sequence coding for extracellular part of IL-17R (IL-17RAex cDNA, Leu33–Trp320, 287 amino acids) was amplified by PCR using forward primer IL17RAex-F-Nco-his ATTACCATGGGCAGCAGCCACCATCATCATCATCACAGCAGCGGCCTGCGACTCCTGGACCACC and reverse primer IL17RAex-R-Bam ATATGGATCCTCACCACAGGGGCATGTAGTCCG. The resulted PCR product was cloned in vector pET-28b using restriction sites NcoI and BamHI. Protein was produced in *E. coli* SHuffle strain (SHuffle^®^ T7 Express Competent *E. coli*, New England Biolabs, Ipswich, MA, USA). Bacterial cells were grown in LB broth with kanamycin (60 µg/L) at 30 °C, protein production was induced by adding 1 mM IPTG after the culture reached the density OD_600_ = 0.6. Cells were cultivated for 4 h at 30 °C or overnight at 18 °C. Cultures were collected by centrifugation, sonicated in TN buffer (50 mM Tris, 150 mM NaCl, pH = 8.0), and the disrupted cells were spun down at 40,000× *g* for 20 min. Supernatant representing the cytosolic extract was used for purification of soluble protein using Ni-NTA. The pelleted inclusion bodies were dissolved in TN buffer containing 8 M urea (50 mM Tris, 150 mM NaCl, 8 M urea, pH = 8.0), purified on Ni-NTA agarose and eluted in EB buffer containing 4 M urea (50 mM Tris, 150 mM NaCl, 250 mM imidazol, 4 M urea, pH = 8.0). Soluble as well as refolded IL-17RA forms were tested in ELISA and used for ribosome display selection.

### 4.4. ABD Library Construction and Ribosome Display Selection

ABD-derived DNA library was generated as described previously [32]. The library was used for the selection of binders by ribosome display [35]. Pre-selection was performed in wells of Polysorp plate (NUNC, Roskilde, Denmark) coated with His_6_-TolA-AVI (Δ ABD clone) followed by blocking with 3% BSA. Unbound ribosomal complexes were used for further selection steps. For the selection, wells of Polysorp plate were coated with bacterial recombinant soluble human IL-17RA receptor or with the receptor refolded from 4 M urea solution in coating buffer, followed by blocking with 3% BSA. After five-round ribosome display selection, transcribed DNA was inserted into pET-28b-TolA-AVI vector by digestion with restriction endonucleases NcoI and XhoI [38] and a resulted plasmid library was used to transform *E. coli* TOP10 host cells.

### 4.5. Production of In Vivo Biotinylated Protein Binders

ARS binders were prepared in the form His_6_-ARS/ARU-TolA-AVI fusion biotinylated proteins produced in *E. coli* BL21 (DE3) BirA strain. Bacterial cells were cultivated in LB medium containing kanamycin (60 µg/mL) and chloramphenicol (30 µg/mL) and for protein biotinylation, 50 µM d-biotin was added, prepared as 5 mM solution in 10 mM bicine buffer pH = 8.3. Protein production was induced by 1.5 mM IPTG after reaching the cell density OD_600_ = 0.6. Culture was harvested 4 h after the induction, then sonicated in TN buffer, centrifuged and subsequently purified on a Ni-NTA-agarose column, finally eluted with 250 mM imidazole.

### 4.6. Screening of IL-17RA-Targeted Binders in ELISA

Polysorp plate (NUNC, Roskilde, Denmark) was coated by IL-17RA recombinant protein (10 µg/mL, produced in *E. coli* SHuffle strain) or 5 µg/mL human IL-17RA-IgG chimera (Recombinant Human IL-17 RA/IL-17 R Fc Chimera Protein, R&D Systems, Minneapolis, MN, USA) in coating buffer (100 mM bicarbonate/carbonate solution, pH = 9.6) at 7 °C overnight. The next day, the plate was washed by PBST (PBS buffer containing 0.05% Tween, pH = 7.4) and blocked by PBSTB (1% BSA in PBST). The samples of bacterial lysates in PBSTB or purified proteins were applied as a series of different dilutions in PBSTB and bound biotinylated ARS proteins were detected using streptavidin-HRP conjugate diluted in the same buffer 1:10,000 (Pierce, Rockford, IL, USA). Results were visualized by enzymatic reaction of HRP with OPD substrate (Sigma-Aldrich, St. Luis, MO, USA) in citrate buffer (3.31% sodium citrate tribasic dihydrate, phosphoric acid until pH = 5.0), reaction was stopped by 2 M sulfuric acid and absorbance at 492 nm was measured.

### 4.7. Sequence Analysis and Clustering of Selected ARS and ARU Variants

DNA constructs of all selected clones expressing full-length His_6_-ARS-TolA-AVI proteins were sequenced. Multiple alignments of amino acid sequences of the ARS variants and the construction of the UPGMA-based similarity tree were performed using the MEGA5 software (Molecular Evolutionary Genetics Analysis, available online: https://www.megasoftware.net).

### 4.8. Modeling of ARS-IL17RA Interactions

The structure of studied ABD variants (ARS004, ARS012, ARS014, ARS019, and ARS043) was modeled using the MODELLER 9v14 suite of programs [49] based on the ABDwt structure (pdb id 1gjt [50]). Amino acid sequences of the ABD variants were aligned with the Clustal Omega program [51]. The structure of IL-17R was obtained from the available crystal structure of the IL-17/IL-17R complex (pdb id 4hsa [42]). The flexible side chain protein-protein docking was performed using a local copy of the ClusPro server [52,53]. The GBSA implicit solvation molecular dynamics simulations were prepared using the OpenMM Zephyr graphical interface [54] with the Amber96 force field, 2 fs time step, temperature 295 K, and water collisional interval of 0.01099 ps. The calculations were performed using the GPU accelerated version of gromacs program [55] collecting geometry every 10 ps.

### 4.9. Competition ELISA

Polysorp plate (NUNC, Roskilde, Denmark) was coated with recombinant IL-17RA (5 µg/mL, recombinant protein produced in *E. coli* SHuffle strain) or IL-17RA-IgG (Recombinant Human IL-17 RA/IL-17 R Fc Chimera Protein, R&D Systems, Minneapolis, MN, USA) in coating buffer at 7 °C overnight and the washed plates were blocked by PBSTB. Human interleukin IL-17A cytokine (Cell Signaling Technology, Danvers, MA, USA) at the constant concentration (10 nM) presented in serially diluted ARS variants in PBSTB as competitors were added. Binding of IL-17A to IL-17RA was detected by a primary polyclonal rabbit anti-human IL-17 antibody (1:1000, Abcam plc., Cambridge, UK) and the secondary anti-rabbit antibody conjugated with HPR (1:2000, Abcam plc., Cambridge, UK).

### 4.10. Fluorescence-Based Thermal-Shift Assay

Protein samples (0.1 mg/mL) in PBS and 5 × Sypro Orange dye (Sigma-Aldrich, St. Luis, MO, USA) were mixed in total volume of 25 µL. Using the real-time PCR Detection System CFX96 Touch (Bio-Rad Laboratories, Hercules, CA, USA), the proteins were incubated in a thermal gradient from 25 to 80 °C with increments of 0.5 °C and with 30 s-hold intervals. The degree of protein unfolding was monitored by the FRET (fluorescence resonance energy transfer) channel that captured the spectral properties of Sypro Orange unfolded protein complexes (excitation wavelength ~470 nm and emission wavelength ~570 nm). The data were analyzed by the CFX Manager software and the melting temperatures were determined using the first derivative spectra.

### 4.11. Flow Cytometry

All binding assays were performed in HEPES buffered salt solution (HBSS buffer; 10 mM HEPES, pH 7.4, 140 mM NaCl, 5 mM KCl) complemented with 2 mM CaCl_2_, 2 mM MgCl_2_, 1% (*w*/*v*) glucose and 1% (*v*/*v*) FCS (cHBSS) in 96-well culture plates (TPP Techno Plastic Products AG, Trasadingen, Switzerland). For ARS binding assay, 2.5 × 10^5^ cells were incubated in 50 mL of cHBSS with or without His_6_-ARS-TolA-AVI clones or His_6_-ABDwt-TolA-AVI negative control (10 mg/mL) for 30 min at 4 °C. The cells were washed with cHBSS and the cell-bound proteins were stained with streptavidin–phycoerythrin (1:400 dilution) for 30 min at 4 °C. Cells were washed, resuspended in 100 mL of HBSS and analyzed by flow cytometry in a BD LSR II instrument (BD Biosciences, San Jose, CA, USA) in the presence of 1 µg/mL of Hoechst 33258. Data were analyzed by FlowJo software (Tree Star, Ashland, OR, USA) and appropriate gatings were used to exclude cell aggregates and dead cells. Binding data were deduced from the mean fluorescence intensities (MFI).

### 4.12. Binding of ARS Proteins to Human Cells Analyzed by LigandTracer Green Line System

HaCaT cells were seeded in a distinct area of the cell dish and were allowed to attach firmly to the surface for at least 24 h. In vivo biotinylated His_6_-ARS-TolA-AVI clones were labeled with Alexa Fluor 488 for 30 min. The cell dish with 3 mL cell culture medium or PBS (for ARS043) was placed into the LigandTracer Green Line (Ridgeview Instruments AB, Uppsala, Sweden) and the difference between the fluorescence intensity of the target cell area and the area opposite to the target cells (background signal) was measured every 70 s by rotating the dish with a detection delay 5 s. After a baseline measurement (30 min), the ARS protein was added gradually in the three (ARS012, ARS014, ARS019, ARS043) or four (ARS043) increasing concentration (3, 10, 90 nM or 3, 10, 30, 70 nM). Each concentration was incubated until the saturation was obtained. Then, the dissociation of the ligand was recorded after replacing the incubation solution with 3 ml fresh medium or PBS. Binding curves were analyzed using the TraceDrawer 1.7.1. evaluation software. All ligand-receptor interactions were analyzed using the “one-to-one” model. For ARS043, signal levels were normalized to 0% at baseline level.

### 4.13. Testing of the Immunomodulatory Potential of ARS Ligands

For the assay, CCD-1070Sk cells (10^4^) were seeded to 24 well plate. After 24 h, 20 ng/mL IL-17A cytokine alone or in the presence of His_6_-ARS-TolA-AVI clones in different concentrations (100, 50, 5, 0.5 ng/mL) were added. After 6, 15, 24, 30, and 48 h, supernatants were collected and levels of Gro-α and IL-6 were measured by Human CXCL1/GRO α DuoSet (R&D Systems, Minneapolis, MN, USA).

### 4.14. Statistical Analysis

Results were expressed as the arithmetic mean ± standard deviation (SD) of the mean. Statistical analysis was performed by one-way ANOVA followed by Dunnett’s post-test, comparing all the samples with the control, using GraphPad Prism 6.0 (GraphPad Software, San Diego, CA, USA). Significant differences are indicated by asterisks (*, *p* < 0.05; **, *p* < 0.01).

## 5. Conclusions

Collectively, this work describes the generation of a collection of unique ABD-derived proteins called ARS ligands that recognize human IL-17RA receptor with a high-binding affinity. These ARS proteins inhibit binding of human IL-17A cytokine to its cognate cell membrane receptor in vitro and exhibit an immunosuppressive potential demonstrated by suppression of Gro-α secretion from IL-17A-activated CCD-1070Sk skin fibroblasts. These ligands can be used as high-affinity non-IgG probes and can be useful in development of anti-IL-17RA-targeted antagonists with a therapeutic potential.

## Figures and Tables

**Table 1:** Sequence comparison of the ARS binders. The non-mutated ABDwt was aligned with the randomized part of the ARS binders selected by ribosome display. Grey boxes indicate the 11 positions at which the residues of albumin-binding domain (ABD) (aa 20–46) were randomized. The non-randomized N-terminal part of ABD (aa 1–19) contain sequence LAEAKVLANRELDKYGVSD.

Protein	20	21	22	23	24	25	26	27	28	29	30	31	32	33	34	35	36	37	38	39	40	41	42	43	44	45	46
**ABDwt**	Y	Y	K	N	L	I	N	N	A	K	T	V	E	G	V	K	A	L	I	D	E	I	L	A	A	L	P
**ARS004**	V	Y	K	-	L	I	N	Y	A	C	P	V	T	W	V	K	W	V	I	D	P	I	L	A	M	L	P
**ARS012**	V	Y	K	-	L	I	N	M	A	L	Y	V	T	G	V	K	P	W	I	D	V	I	L	A	V	L	P
**ARS014**	V	Y	K	N	L	I	N	Y	A	W	L	V	M	W	V	-	-	P	I	D	A	I	L	A	L	L	P
**ARS019**	M	Y	K	N	V	I	N	I	A	W	W	V	S	I	V	K	Y	P	I	D	C	I	L	A	L	L	P
**ARS043**	L	Y	K	N	M	I	N	M	A	L	W	V	T	G	V	K	W	L	I	D	P	I	L	A	T	L	P

## Supplementary Materials

Supplementary materials can be found at http://www.mdpi.com/1422-0067/19/10/3089/s1.
