# Supplementary material for: ABD-Derived Protein Blockers of Human IL-17 Receptor A as Non-IgG Alternatives for Modulation of IL-17-Dependent Pro-Inflammatory Axis
Source: Int J Mol Sci. 2018 Oct 9;19(10):3089. doi: 10.3390/ijms19103089 (PMC6213189; doi:10.3390/ijms19103089)
Supplement: Supplementary file 1 [file ijms-19-03089-s001.pdf]

ABD-derived protein blockers of human IL-17 receptor A as non-IgG alternatives for modulation of IL-17-dependent pro-inflammatory axis

Marie Hlavničková, Milan Kuchař, Radim Osička, Lucie Vaňková, Hana Petroková, Michal Malý, Jiří Černý, Petr Arenberger and Petr Malý

Supplementary Data

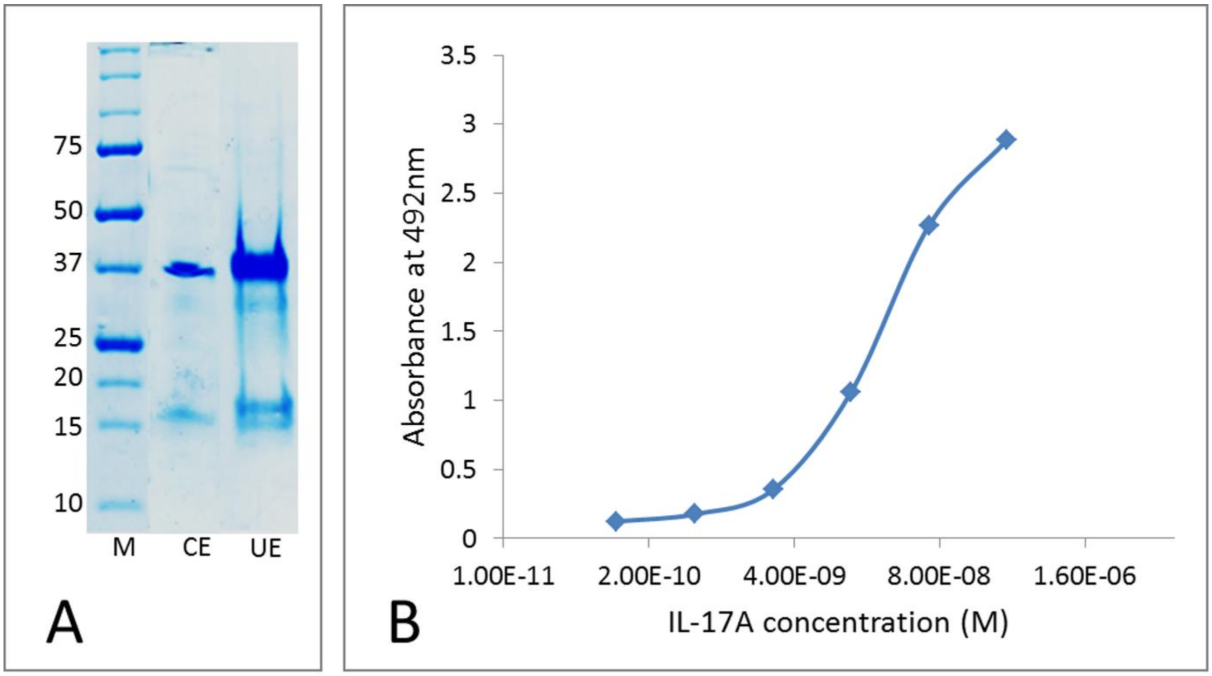

Figure S1. Production and functional verification of the recombinant IL-17RA receptor. (A) The recombinant IL-17RA protein was produced in *E. coli* SHuffle strain, purified on Ni-NTA-agarose chromatography and the purity of the protein was verified on SDS-PAGE (CE indicates a soluble protein purified from a cytosolic fraction, UE indicates protein purified from an urea extract). Recombinant product of 40 kDa was visualized on polyacrylamide gel. (B) Binding activity of recombinant IL-17RA produced in *E. coli* tested in ELISA. The protein purified on Ni-NTA agarose, eluted in 4 M urea and diluted in a coating buffer was immobilized in 96-well plate overnight. The human IL-17A protein was added in series of dilutions in phosphate buffer and its binding was detected using anti-human IL-17A rabbit polyclonal antibody followed by anti-rabbit IgG-HRP conjugate.

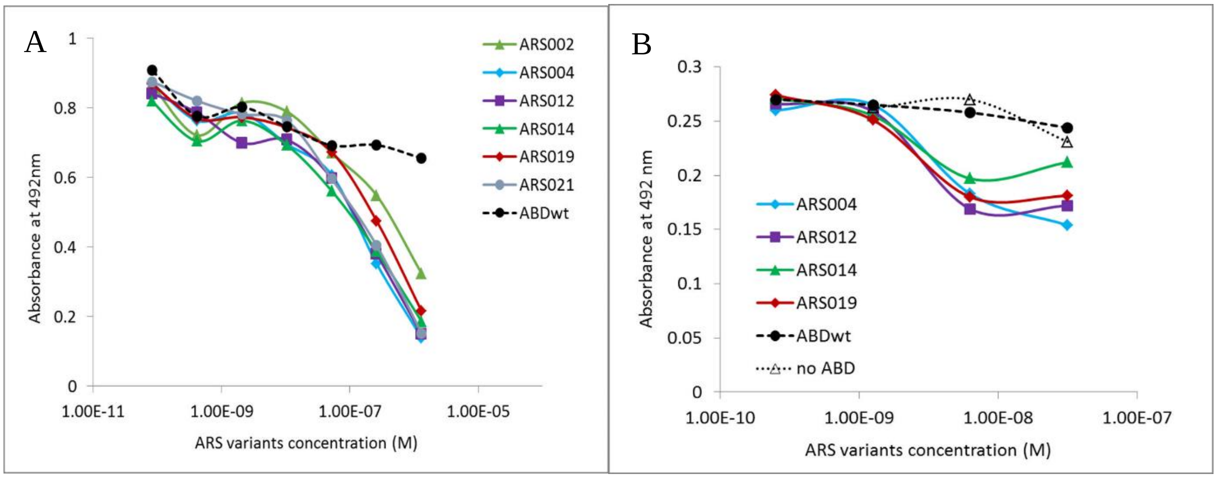

Figure S2. ARS ligands compete with IL-17A cytokine for binding to human recombinant IL-17RA (A) or IL-17RA-IgG chimera (B) in ELISA. IL-17RA produced in *E. coli* SHuffle host cells was immobilized on 96-well Polysorp plate. Serially diluted His_6-ARS-TolA-AVI ligands were used to compete for binding with 10 nM of human IL-17A cytokine. Bound IL-17A was detected with anti-human IL-17A rabbit polyclonal antibody in combination with secondary anti-IgG-HRP conjugate. His_6-ABDwt-TolA-AVI served as a negative control.

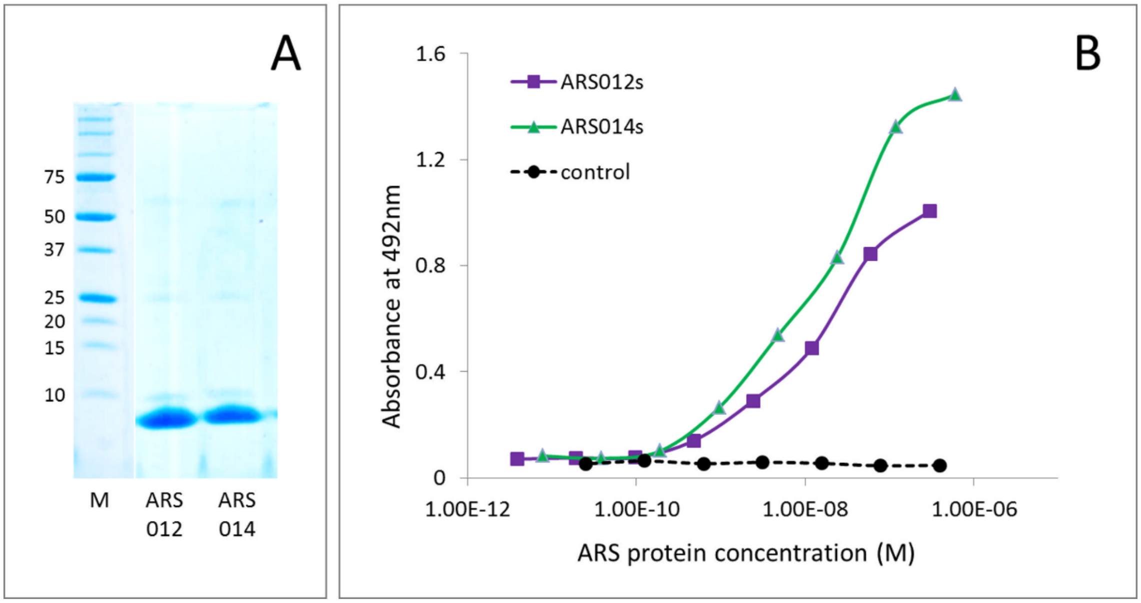

Figure S3. Generation and characterization of short ARS proteins. (A) Production of *in vivo* biotinylated His_6-ARS-AviTag protein variants shown as stained bands of 8.3 kDa Ni-NTA-agarose purified proteins on SDS-PAGE. (B) Binding of biotinylated ARS012s and ARS014s proteins to immobilized recombinant IL-17RA, produced in *E. coli* SHuffle host cells, tested in ELISA using streptavidin-HRP conjugate. As a negative control, coated BSA in the absence of IL-17RA was used.

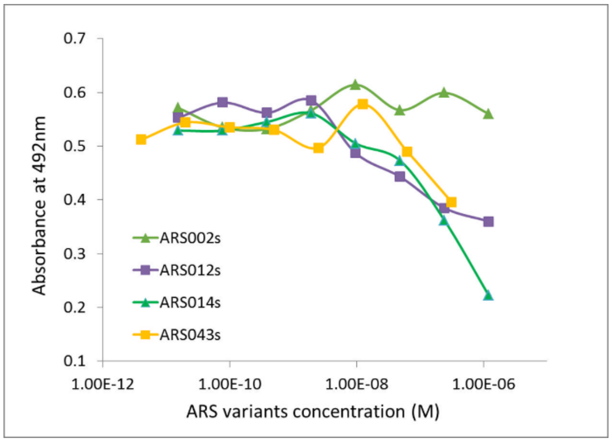

Figure S4. Short versions of ARS ligands inhibit binding of human IL-17A cytokine to recombinant IL-17RA. Serially diluted ARS proteins in PBSTB buffer competed with 10 nM IL-17A for binding to *E. coli*-produced IL-17RA. Bound IL-17A was detected with anti-IL-17A polyclonal antibody in combination with secondary anti-IgG-HRP conjugate.

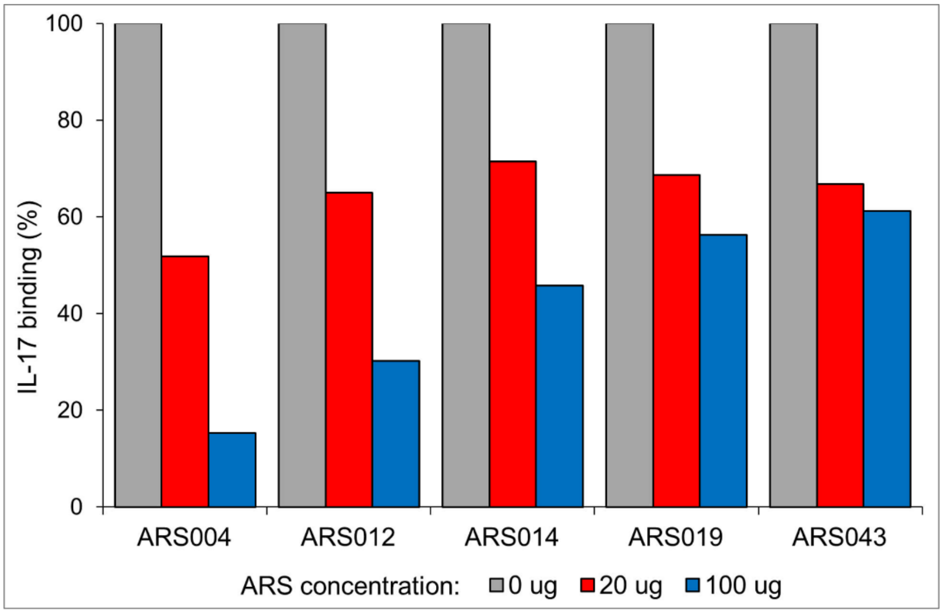

Figure S5. ARS ligands inhibit binding of IL-17 to THP-1 cells. THP-1 cells (2x10$^{5)}$ expressing IL-17RA were pre-incubated without or with two different concentrations (20 and 100 µg/ml) of particular ARS proteins (30 min at 4°C) and incubated with 10 µg/ml of IL-17 (30 min at 4°C). After washing, the surface-bound IL-17 was stained with anti-IL-17 rabbit antibody (30 min at 4°), washed and after the incubation with a Cy5-labeled goat anti-rabbit IgG (30 min at 4°C) analyzed by flow cytometry. Binding of IL-17 to ARS-untreated THP-1 cells was taken as 100%.

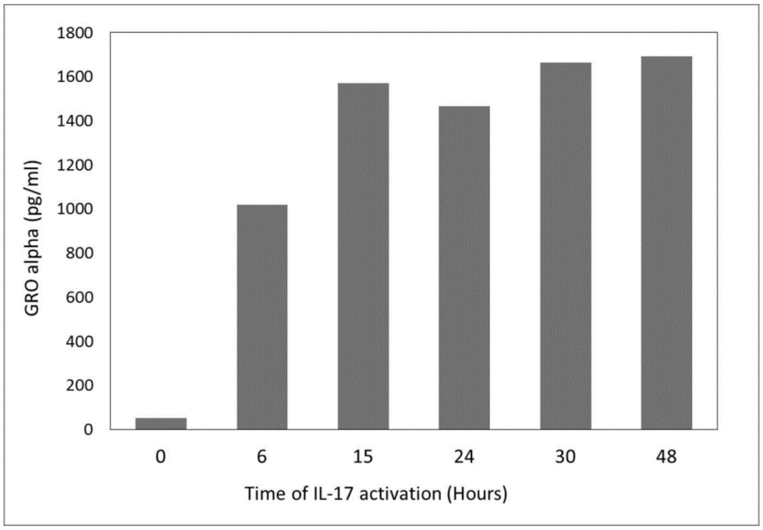

Figure S6. Secretion of Gro-α by CCD-1070Sk skin fibroblasts upon stimulation by IL-17A. Cells were stimulated by 20 ng/ml human IL-17A cytokine and after cultivation for 6, 15, 24, 30 or 48 hours, cell supernatants were collected and the levels of Gro-α were measured by ELISA.
